# Editorial: Brain abnormalities due to genetic alterations or developmental exposure to environmental factors

**DOI:** 10.3389/fnins.2022.944861

**Published:** 2022-07-26

**Authors:** Kazuhiko Sawada, Atsushi Yoshiki, Shigeyoshi Saito

**Affiliations:** ^1^Department of Nutrition, Faculty of Medical and Health Sciences, Tsukuba International University, Tsuchiura, Japan; ^2^Experimental Animal Division, RIKEN BioResource Research Center, Tsukuba, Japan; ^3^Division of Health Sciences, Department of Medical Physics and Engineering, Graduate School of Medicine, Osaka University, Suita, Japan; ^4^Department of Advanced Medical Technologies, National Cerebral and Cardiovascular Center, Research Institute, Suita, Japan

**Keywords:** neurodevelopmental disorders, brain, cerebral cortex, cerebellum, neurogenesis, maldevelopment, epigenesis

The structural and functional development of the brain is altered by many genetic and environmental factors, including drugs, industrial chemicals, nutrition, infections, irradiation, prenatal stress, and maternal separation. These factors can influence various neurodevelopmental events, such as neurogenesis, neural differentiation, neural migration, apoptosis, axonal connections, and synaptogenesis, which can be triggered directly or *via* epigenetic dysregulation and can often cause irreversible changes in the brain. This Research Topic on “*Brain Abnormalities due to Genetic Alterations or Developmental Exposure to Environmental Factors*” comprises 12 papers published in Frontiers in Neuroscience, including three review articles and nine original articles focusing on the brain development abnormalities caused by various genetic and environmental factors.

Many studies in this research area have focused on the structural and functional maldevelopment of the brain in animal models for neurodevelopmental disorders. These papers emphasize multifaceted perspectives on maldevelopmental changes in the brain common to different neurodevelopmental disorders, studied using various research strategies. A review by Uchida and Suzuki mentioned the histoarchitecture and altered chemical phenotypes of neurons in the cerebrum in congenital hypothyroidism. The decreased parvalbumin expression in cortical neurons, shown in several mouse models of hypothyroidism, was reminiscent of the maldevelopment of cortical phenotypes in autism spectrum disorder (ASD) and schizophrenia. Original articles by Sethi et al. and Stietz et al. reveal that the behavioral characteristics and dendritic morphogenesis in the cerebrum were altered depending on the sex and neuronal Ca^2+^ signaling-related gene variants in mice with developmental exposure to an endocrine disruptor, polychlorinated biphenyl (PCB). Since developmental PCB exposure confers a risk of neurodevelopmental disorders, including ASD and attention deficit hyperactivity disorder (ADHD), individual variabilities in genetic characteristics may modify the clinical phenotypes of these neurodevelopmental disorders. An original article by Zhou et al. reported that social interaction deficits were improved by modulating vasopressin pathways in ASD model rats developmentally exposed to valproic acid (VPA). These findings indicate the possibility of a clinical application of arginine vasopressin treatment to improve behavioral symptoms in patients with ASD. A review article by Ossola and Kalebic cited the involvement of subventricular zone (SVZ) progenitors in the malformation of cortical development in neurodevelopmental disorders. In particular, the proliferation and polarity of progenitors are key factors that are disrupted in the malformation of cortical development in gyrencephalic mammals. This hypothesis is supported by previous studies, which showed that manipulations of SVZ progenitor proliferation alter cortical folding (Rabe et al., 1985; Masuda et al., 2015; Sawada et al., 2021). An original article by Sawada et al. reinforces the supposition that the modification of neuronal progenitor proliferation is involved in brain maldevelopment. They revealed that the fate of hippocampal dentate gyrus progenitors was manipulated by developmental exposure to VPA. This study is unique in that it used ferrets as experimental animals as they are characterized by slow development and maturation of the brain, sustaining cerebral and cerebellar neurogenesis until early postnatal life, which is reminiscent of humans. The findings, along with other studies (Fujimura et al., 2016; Lauber et al., 2016; Sawada et al., 2021) facilitate a comprehensive understanding of brain maldevelopment in VPA-exposed ASD models. In an epidemiological study conducted by Deng et al., factors contributing to ADHD susceptibility have been epidemiologically identified in preschool-aged children in Beijing and Tangshan City. Notably, this study indicated that parental influences such as smoking, breastfeeding, and patience, during the prenatal and early postnatal periods, may increase the risk of ADHD susceptibility.

Other studies in this Research Topic focus on the impact of particular molecules or genes on brain maldevelopment. A review article by Hawkes summarizes the deformations of the cerebellar zone-and-stripe patterns in mice with spontaneous gene mutations or gene manipulations. We can study the cerebellar zone-and-stripe development that is organized by the interplay of two distinct developmental pathways, one for inhibitory Purkinje neurons and the second for excitatory granular neurons, by focusing on particular genes. An original article by Shabanipour et al. showed two alternative suppositions for the roles of neural cell adhesion molecule 1 in the inhibitory Purkinje cell developmental pathway. One is excessive Purkinje cell migration along the Bergmann glia, and the other is Purkinje cell monolayering. Original articles by Sun et al. and Wang et al. assessed the effect of gene knockout for *Plppr5*, a subclass of the lipid phosphate phosphatase superfamily, on developmental seizure-induced late-onset hypersensitivity and hypoxia-ischemia-induced cortical damage with long-term excitability. These changes may be mediated by the disruption of zinc- and mitochondria-dependent metabolic pathways. These findings suggest that different environmental stimuli for specific genes during development can result in diverse brain abnormalities. An original article by Tian et al. reported primary visual cortex atrophy with the reorganization of functional connectivity accompanied by strengthened connectivity to the default mode network in patients with chronic Leber's hereditary optic neuropathy. This report placed a focus on hereditary diseases carrying mutations in mitochondrial DNA in terms of “Brain Abnormalities due to Genetic Alterations,” which is one of the themes of this Research Topic.

The articles on “models for neurodevelopmental disorders” and “impacts of particular molecules and/or genes” in brain maldevelopment have facilitated our understanding of the themes of the Research Topic, “*Brain Abnormalities due to Genetic Alterations or Developmental Exposure to Environmental Factors*.” We hope that these findings will provide readers with new insights and baselines for investigating the normal and abnormal development of mammalian brain structures and functions.

## Author contributions

All authors listed have made a substantial, direct, and intellectual contribution to the work and approved it for publication.

## Conflict of interest

The authors declare that the research was conducted in the absence of any commercial or financial relationships that could be construed as a potential conflict of interest.

## Publisher's note

All claims expressed in this article are solely those of the authors and do not necessarily represent those of their affiliated organizations, or those of the publisher, the editors and the reviewers. Any product that may be evaluated in this article, or claim that may be made by its manufacturer, is not guaranteed or endorsed by the publisher.
